# Subgroups of lumbo-pelvic flexion kinematics are present in people with and without persistent low back pain

**DOI:** 10.1186/s12891-018-2233-1

**Published:** 2018-08-28

**Authors:** Robert A. Laird, Jennifer L. Keating, Peter Kent

**Affiliations:** 1 Department of Physiotherapy, Monash University, PO Box 527, Frankston, Victoria 3199 Australia; 2 School of Physiotherapy and Exercise Science, Curtin University, Perth, Australia; 3 Department of Sports Science and Clinical Biomechanics, University of Southern Denmark, Odense, Denmark; 4 Superspine, Forest Hills, Melbourne, Australia

**Keywords:** Low back pain, Subgroups, Patterns, Movement disorders, Range of movement (ROM), Flexion relaxation, Lumbo-pelvic rhythm, Velocity

## Abstract

**Background:**

Movement dysfunctions have been associated with persistent low back pain (LBP) but optimal treatment remains unclear. One possibility is that subgroups of persistent LBP patients have differing movement characteristics and therefore different responses to interventions. This study examined if there were patterns of flexion-related lumbo-pelvic kinematic and EMG parameters that might define subgroups of movement.

**Methods:**

This was a cross-sectional, observational study of 126 people without any history of significant LBP and 140 people with persistent LBP (*n* = 266). Wireless motion and surface EMG sensors collected lumbo-pelvic data on flexion parameters (range of motion (ROM) of trunk, lumbar, and pelvis), speed, sequence coordination and timing, and EMG extensor muscle activity in forward bending (flexion relaxation)), and sitting parameters (relative position, pelvic tilt range and tilt ratio). Latent class analysis was used to identify patterns in these parameters.

**Results:**

Four subgroups with high probabilities of membership were found (mean 94.9%, SD10.1%). Subgroup 1 (*n* = 133 people, 26% LBP) had the greatest range of trunk flexion, fastest movement, full flexion relaxation, and synchronous lumbar versus pelvic movement. Subgroup 2 (*n* = 73, 71% LBP) had the greatest lumbar ROM, less flexion relaxation, and a 0.9 s lag of pelvic movement. Subgroup 3 (*n* = 41, 83% LBP) had the smallest lumbar ROM, a 0.6 s delay of lumbar movement (compared to pelvic movement), and less flexion relaxation than subgroup 2. Subgroup 4 (*n* = 19 people, 100% LBP) had the least flexion relaxation, slowest movement, greatest delay of pelvic movement and the smallest pelvic ROM. These patterns could be described as standard (subgroup 1), lumbar dominant (subgroup 2), pelvic dominant (subgroup 3) and guarded (subgroup 4). Significant post-hoc differences were seen between subgroups for most lumbo-pelvic kinematic and EMG parameters. There was greater direction-specific pain and activity limitation scores for subgroup 4 compared to other groups, and a greater percentage of people with leg pain in subgroups 2 and 4.

**Conclusion:**

Four subgroups of lumbo-pelvic flexion kinematics were revealed with an unequal distribution among people with and without a history of persistent LBP. Such subgroups may have implications for which patients are likely to respond to movement-based interventions.

**Electronic supplementary material:**

The online version of this article (10.1186/s12891-018-2233-1) contains supplementary material, which is available to authorized users.

## Background

Persisent low back pain (LBP) is often described as a multidimensional problem, within a bio-psycho-social context [1, 2]. Dimensions that are thought to influence pain and function include patho-anatomic changes, cognitions and emotions, lifestyle, societal circumstances, and movement/posture [3–9]. People with LBP are quite heterogeneous within these dimensions. Identifying clinically important subgroups that are relatively homogenous within these dimensions has been a research priority [10, 11], based on a prevailing belief that better outcomes are likely when treatment is matched with subgroup-specific features.

A number of movement-based classification systems have been developed, underpinned by observations of relationships between movement and LBP, with the intention of providing subgroup-specific, targeted treatment [8, 12–15]. Different classification systems use different, albeit overlapping, combinations of examination findings to define subgroups, [16]. Examination findings include subjective reports, visual observation and pain responses to movement, but rarely include measurement of lumbo-pelvic kinematic parameters.

There is evidence that flexion-related activities are particularly important in LBP. For example, in a study on people with subacute LBP by Pengel et al. [17], the three most frequently nominated pain-related activities were sitting, bending and lifting, which all involve elements of flexion. As a consequence, there are potentially important clinical questions to be investigated in empirical measurements of flexion-related lumbo-pelvic kinematics: (i) are there different patterns in the way people perform flexion, and (ii) are any patterns more common in people with persistent LBP than in people who have never had LBP?

Studies of lumbo-pelvic kinematic parameters have identified differences in range of motion (ROM) in people with and without LBP, using between-group mean differences and their standard deviations (SD), but have generally not described subgroups based on lumbo-pelvic kinematics [18, 19]. Identifying that lumbar ROM is, on average, reduced in people with LBP [18] would suggest that improving ROM might be a treatment target. However, if some people with LBP do not have reduced lumbar ROM, a treatment strategy aimed at increasing lumbar ROM may be unhelpful. Lumbo-pelvic kinematics include a range of parameters such as trunk, lumbar and pelvic ROM, timing of regional movement, muscle activation, movement duration, movement coordination, and postural position. Using multivariable clusters of these kinematic parameters may identify different patterns of flexion that might assist in matching targeted interventions to specific lumbo-pelvic kinematic goals.

Previous work by Marras et al. [20], Dankaerts et al. [21] and Mayer et al. [22] all used kinematic analysis to validate pre-defined subgroups of people with persistent LBP but did not use kinematic data a priori to define subgroups. Marras et al. [20] quantified and matched angular data, velocity and acceleration kinematic parameters to modified Quebec classification subgroups. Dankaerts et al. [21] measured ROM and EMG parameters in two subgroups of people classified with an O’Sullivan classification system [14] and Mayer et al. [22] pre-classified people with persistent LBP into four groups based on ‘normal’ versus ‘abnormal’ lumbo-pelvic ROM and EMG of lumbar extensors during flexion.

The availability of wireless inertial and EMG sensors for use in clinical environments now enables detailed and accurate measurement of lumbo-pelvic movement. A recent study (Laird et al., 2018, unpublished) on lumbo-pelvic kinematics using data from this type of device found that, compared to people without LBP, people with persistent LBP showed a higher prevalence of smaller trunk, lumbar and pelvic ROM, slower movement, delayed pelvic versus lumbar movement and greater lumbar extensor muscle activation in the fully flexed position. That study also identified a wide range of variance for most parameters. It did not, however, investigate whether subgroups of movement patterns were evident in the data.

The current study aimed to explore (i) if patterns (subgroups) of flexion-related lumbo-pelvic kinematics could be identified in a suitably large sample of people, (ii) if patterns were present, whether they occurred with different frequency in people with and without persistent LBP, and (iii) to investigate clinical and demographic characteristics that are associated with any patterns.

## Method

This cross-sectional, observational study used latent class analysis to identify subgroups in the movement patterns of flexion-related lumbo-pelvic kinematics using a previously reported dataset (Laird et al. 2018).

### Study sample

Inclusion and exclusion criteria have been previously reported in detail [23]. In summary, 140 adults (18–65 years old) with persistent LBP were recruited from primary and secondary care (physiotherapy clinics and outpatient departments). Inclusion criteria were LBP > 3 months’ duration, pain scores of 3 or higher (on a 0–10 point numerical rating scale), with current back +/− leg pain. Exclusion criteria were previous lumbar surgery; any invasive spinal procedures for LBP, including therapeutic injections, within the last 12 months; any serious medical or musculoskeletal issues that had the potential to affect the lumbo-pelvic region; an implanted electrical medical device; a BMI > 30 (where it becomes difficult to palpate bony landmarks); or pregnancy. Adults (*n* = 126) who had never had LBP (NoLBP group) were recruited from universities, workplaces and community groups by poster and word of mouth advertising and were eligible for inclusion if they had no significant health issues that would affect movement, and no history of any LBP episode that required visiting a health professional or taking time off either work or usual sport. All participants were screened for inclusion and exclusion initially by administrative staff and then re-checked by the assessing clinician. In addition, people in the NoLBP group were asked if they had any current LBP and excluded if they did. Demographic data can be seen in Table 1. There was a significant difference in age between the groups, as people with in the LBP group were, on average, 7 years older than those in the NoLBP group.

**Table 1:** Between-group comparisons for demographic and kinematic data

Demographics	Details	NoLBP (*n* = 124)	LBP (*n* = 140)	*p*-value
Age (years)		34.4 ± 13.5^a^	41.4 ± 12.6	*p* *= .0001*^b^
BMI		23.6 ± 3.5	25.6 ± 4.9	*p* = .*0001*^b^
Sex - *% female*		59%	57%	*p* = .8250
Pain intensity (0–10)			5.3 ± 1.5	not applicable
Activity limitation (0–100)			39 ± 21	not applicable
Kinematic parameters		No LBP (*n* = 124)	LBP (*n* = 140)	*p*-value
Flexion: Peak trunk flexion	Trunk flexion angular inclination (T12)	111^o^ ± 16^o^	93^o^ ± 16^o^	*p* *< .0000*^b^
Flexion: Peak lumbar flexion	Lumbar ROM	52^o^ ± 11^o^	46^o^ ± 12^o^	*p* *< .0000*^b^
Flexion: Peak pelvic flexion	Pelvic flexion angular inclination (S2)	59^o^ ± 15^o^	48^o^ ± 15^o^	*p* *< .0000*^b^
Flexion: Lumbo-pelvic co-ordination	Mean Lumbar % contribution	48 ± 11%	49 ± 11%	*p* = .217
Flexion: Flexion Relaxation Response	A ratio formed by units of surface EMG activity	0.012 ± 0.32	0.25 ± 0.32	*p* *< .0000*^b^
Sitting: Mean pelvic tilt range	Range from full anterior tilt to full posterior tilt	29^o^ ± 13^o^	29^o^ ± 13^o^	*p* = .883
Sitting: Mean pelvic tilt ratio	A ratio of pelvic tilt range/range of trunk ROM change	2.1 ± 1.3	2.4 ± 1.4	*p* = .064
Sitting: Mean relative sitting position	Max slump sit = 100%, maximum upright sit = 0%	48 ± 35%	50 ± 35%	*p* = .619
		No LBP (*n* = 100)	LBP (*n* = 105)	
Flexion: Delay at 0^o^	Mean delay (negative numbers indicate pelvic delay)	−0.21 ± 0.46 s	−0.36 ± 0.46 s	*p* *= .023*^b^
Flexion: Delay at 20^o^	Mean delay (negative numbers indicate pelvic delay)	−0.30 ± 0.88 s	−0.51 ± 0.90s	*p* = .105
Flexion: Mean movement duration	Time from start of flexion to full flexion	2.28 ± 0.94 s	3.18 ± 0.94 s	*p* *< .0000*^b^

^a^All data represented as mean and standard deviation ^b^significant *p* values italicised

### Data collection

Data were collected on age, sex, BMI, and for people with persistent LBP only, pain intensity (numerical rating scale 0–10 using the average of current, usual, and worst pain scores) [24], activity limitation (Roland Morris Disability Questionnaire) [25] and a study-specific, non-validated ‘does flexion aggravate and extension ease’ (FLAG) pain questionnaire. The FLAG is scored from 0 to 48 where higher scores indicate a greater pattern of flexion-aggravating and extension-easing pain behaviour (see Appendix). The FLAG has four questions, two that ask about flexion-aggravating activities and two that ask about extension-easing activities. Each question has two parts: the first part asks about frequency and is scored (a) never =0, rarely =1 sometimes =2, often =3, always =4; and the second part asks about intensity and is scored none =0, low =1, medium =2, and high =3. For each of the four questions, a score is calculated by multiplying frequency (0–4) by intensity responses (0–3) with possible scores of 0–12. Scores for the four questions were then summed to give an indication of the extent to which flexion aggravated and extension eased pain (maximum score = 48).

Movement data were collected using wireless inertial motion and electromyographic (EMG) sensors (ViMove hardware and software, DorsaVi, Melbourne, Australia). Participants were partially undressed, without shoes and stood in a relaxed upright position. Motion sensors were placed over T12 and S2, and EMG sensors applied 1.5 cm either side of L3, using a standardized procedure. Motion sensors were calibrated to zero in the relaxed standing position.

### Movements analysed

**Fig. 1:**
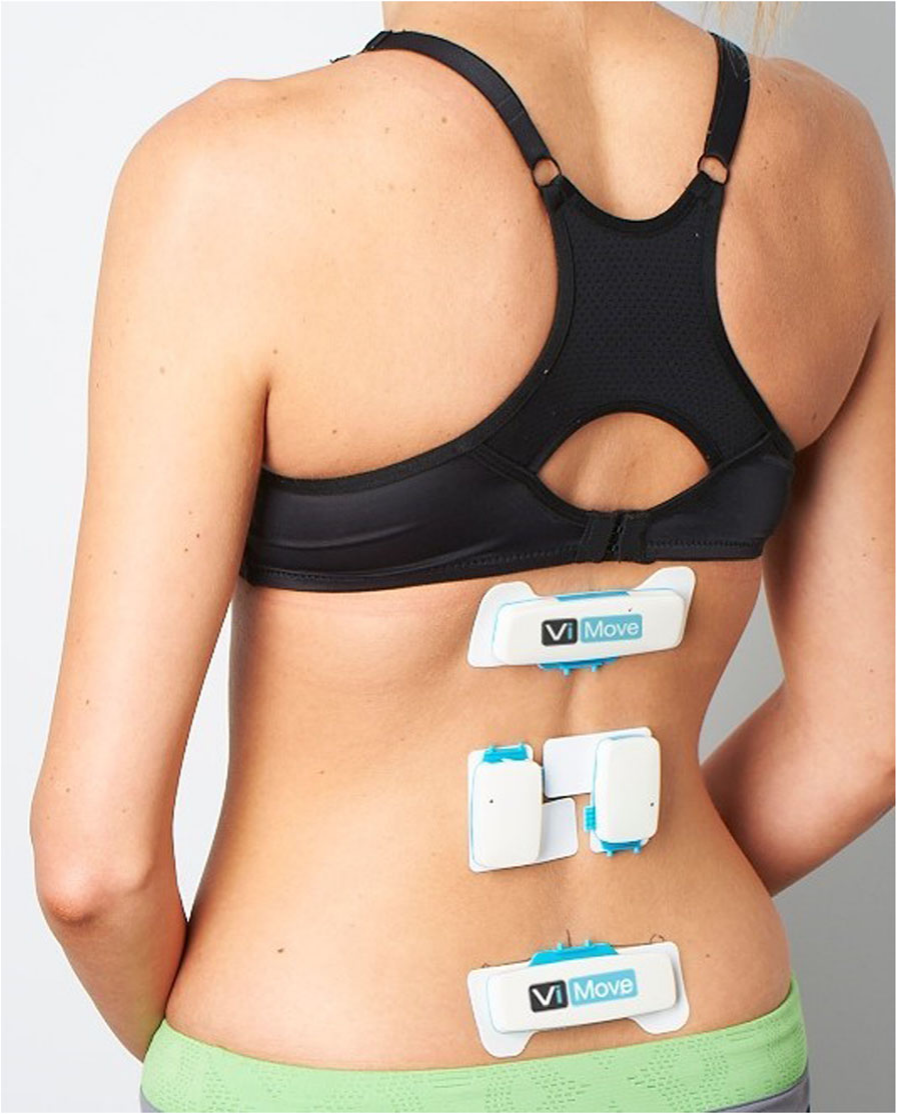
Sensor placement

Movement and positional data were recorded for standing, flexion and sitting. People were asked to stand in their normal standing pose. They were then asked to bend (flex) towards the ground as far they could. A single practice repetition was performed. Three repetitions of flexion with a time count of 3 s in the fully flexed position were then performed, using standardized instructions from trained testers and were automatically captured by a computerized process. Patients were then instructed to sit in their usual, full slumped and full upright sitting positions with angular inclination data averaged over 5 s for each position once the position was stable. Figure 1 demonstrates the sensor placement.

### Lumbo-pelvic kinematic parameter definitions

Eight flexion lumbo-pelvic kinematic parameters were assessed during a standing flexion movement including (i) trunk ROM (angular inclination of the trunk at T12), (ii) pelvic ROM (angular inclination of the pelvis at S2), each measured as maximum angular displacement, (iii) lumbar ROM measured as the difference between trunk angular displacement at T12 and pelvic angular displacement at S2, (iv) lumbo-pelvic coordination (also known as lumbo-pelvic rhythm) measured as the percentage of lumbar contribution to trunk movement, using two methods; area under the curve and peak angular displacement, (v) the flexion relaxation response (a response where lumbar extensors muscles show full relaxation in the fully flexed position in healthy individuals [26]) measured as summed EMG activity of extensor muscle activity during the fully flexed position divided by the sum of EMG activity during eccentric (standing to full flexion) and concentric (return from full flexion) phases (vi) the duration/time of eccentric flexion from the start of movement to full flexion where the beginning and end of the movement was determined by a velocity of > 7°/sec then < 7°/sec respectively, (vii and viii) relative timing of lumbar versus pelvic movement at the beginning of the movement and at 20° (i.e did both lumbar and pelvic regions move synchronously or was there a time-related delay in the movement of lumbar or pelvic regions at the onset of movement, or in the time it took for each region to achieve 20° of flexion).

The three sitting kinematic parameters included (i) pelvic tilt range, the difference between full posterior and full anterior pelvic tilt as measured by angular inclination at S2, (ii) a ‘pelvic tilt ratio’ which compared the amount of angular pelvic tilt movement to angular tilting at T12, where numbers > 1 indicate more pelvic than trunk movement and numbers < 1 indicate more trunk than pelvic movement and (iii) the ‘usual’ sitting position, a relative sitting position, calculated as a percentage where the slumped sitting angle (full posterior pelvic tilt) was 100% and the angle of upright sitting (full anterior tilt) was 0%. These parameters are described in detail in Additional file 1.

A summary of results for flexion and sitting can be seen in Table 1 at a group level. Due to a software version evolution between 2011 and 2014, the time related and sitting variables were only available for people measured after 2014 (LBP group = 105 and NoLBP = 100), whereas the range of movement and EMG-related data, were available for all participants.

### Statistical analyses

Latent Class Analysis, a probabilistic form of unsupervised (data-driven) analysis, was used to identify potential subgroup models. Latent Class models were estimated for up to 10 subgroups, using 500 random seed points to reduce the possibility of local solutions. A co-variate consisting of the LBP/NoLBP status of each participant was included in each model to assist in post-hoc analysis but did not contribute to the subgroup modelling. The resultant models were examined for the degree of contributions of each kinematic variable and residual correlations within classes. Model fit was assessed using the Bayesian Information Criterion and informed by posterior probability diagnostics (average posterior probability for each subgroup, classification error and odds of correct classification). We planned to choose the model with the lowest Bayesian Information Criterion score, provided it reduced the criterion score by 1% or more when adding a subgroup [7]. Indicator variables that were not contributing to the discrimination of subgroups (*r*^2^ < 10%) were removed to create more parsimonious models that estimated fewer parameters and had more power. After the final model was chosen, participants were assigned to subgroups based on their individual posterior probability.

A post-hoc analysis of between-subgroup differences was performed, to assist in profiling and subgroup description. For variables that were normally distributed, a one-way analysis of variance was used with post-hoc (unadjusted alpha level *p* = .05, Bonferroni adjusted alpha level *p* = 0.0083) t-test pairwise comparisons. For variables that were not normally distributed, a Kruskal–Wallis Test was used followed by Dunn’s test for pair-wise (Bonferroni adjusted alpha level *p* = .0083) comparisons. Latent Class Analysis was undertaken using Latent GOLD 4.5 (Statistical Innovations Inc., Belmont, CA, USA) and all other statistical procedures used Stata/IC version 15 (StataCorp, College Station, TX, USA).

### Ethics

Ethics approval was obtained from the Monash University Human Research Ethics Committee (approval number 2016–1100) and from the Regional Committees on Health Research Ethics for Southern Denmark (approval number S-20110071). All participants were given information about the study and they provided written informed consent.

## Results

### Selection of subgroups

Initially, latent class models included all 11 kinematic variables but, as the sitting-related variables all contributed little to the subgroup models (all with an r^2^ < 4% for each variable), we subsequently removed mean pelvic tilt range, pelvic tilt ratio and usual sitting position from further model building. The model with the lowest eligible Bayesian Information Criterion score, was the four-subgroup model. The mean (SD) probability of membership for subgroups 1 to 4 was 95.1% (10.0%), 91.2% (13.4%), 96.7% (7.7%) and 96.6% (11.1%) respectively, which were considerably above the recommended minimum for model adequacy of 70% [27]. Collectively, 92.6% of participants had a posterior probability of > 80.0% of belonging to the subgroup into which they were classified and 84.0% of participants had a greater than 90.0% probability. The overall classification error of the four-subgroup model was acceptable at 5.6%.

**Fig. 2:**
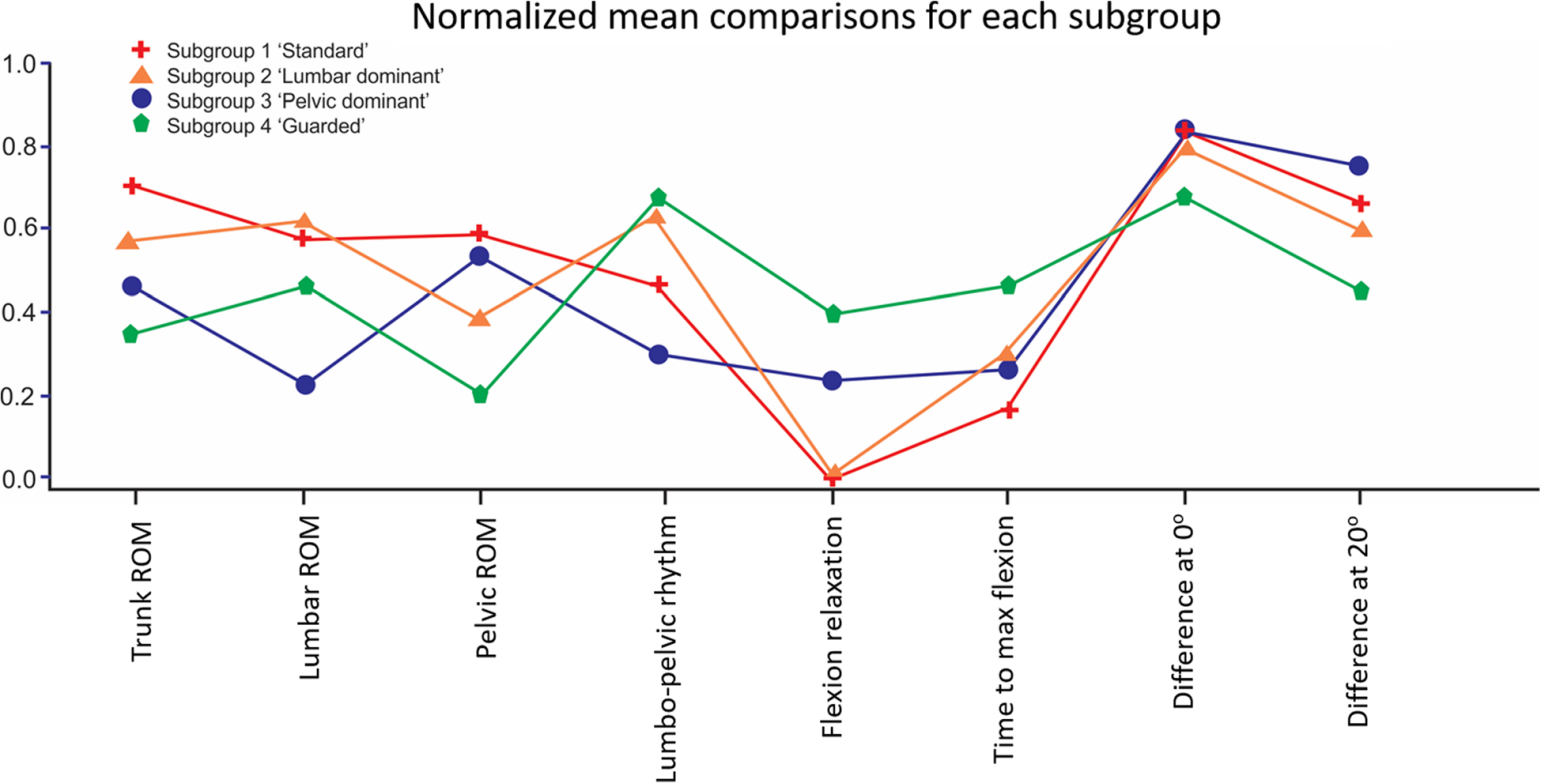
Comparisons of the means for each subgroup on each kinematic parameter (scale normalised to 0–1). Figure 2 illustrates a clinical visualization for each subgroup, with angular inclination for trunk (at T12), pelvis angular inclination (at S2), lumbar movement range and lumbar extension muscle activity (with movement duration and pelvic or lumbar delay at 20^o^ added as text below each subgroup). On the normalised scale of 0–1, 0 is the lowest score observed and 1 is the highest score

**Fig. 3:**
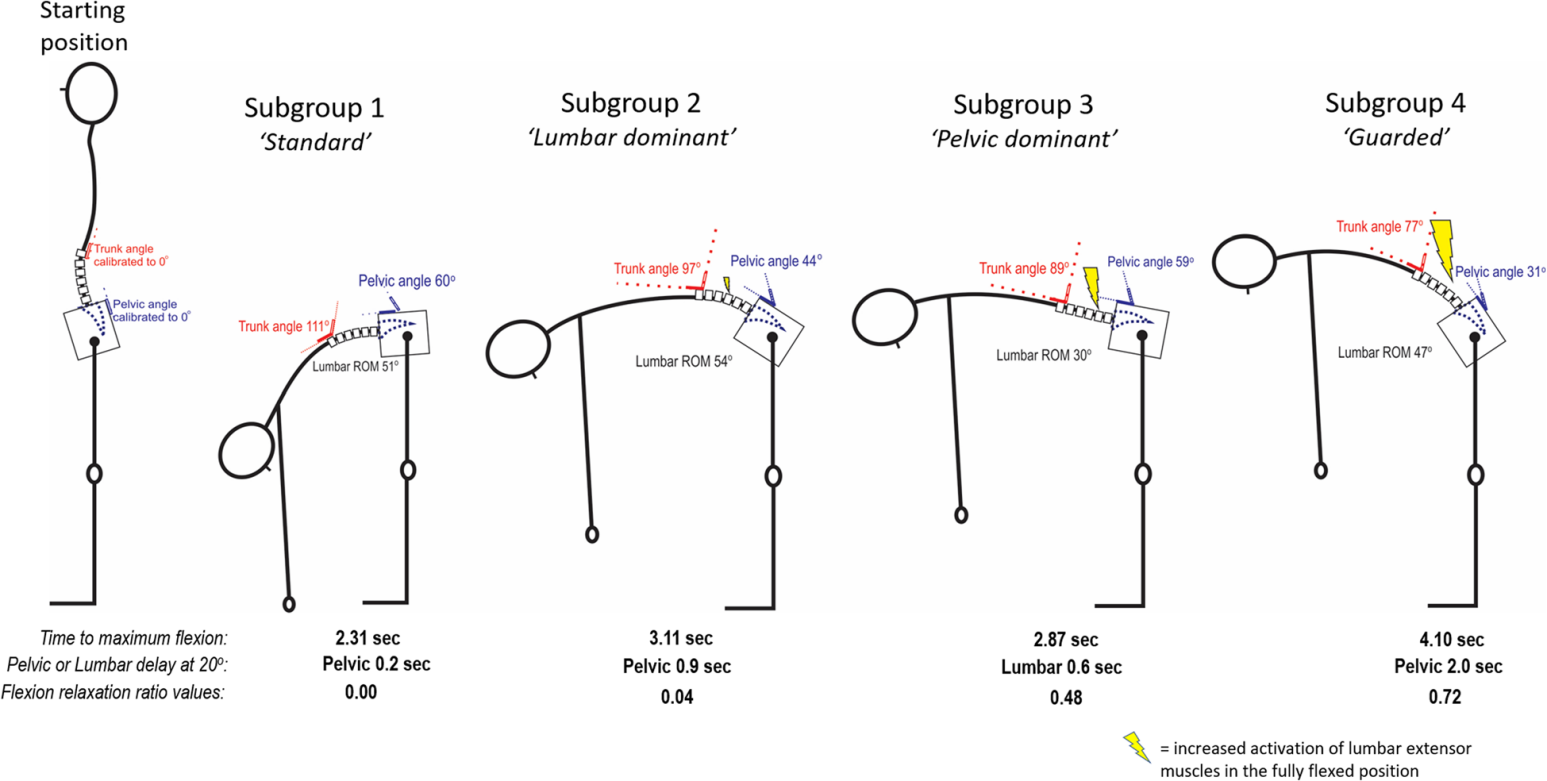
Clinical visualization of mean peak kinematic parameters, temporal and muscle relaxation parameters for each subgroup. This figure illustrates the four-subgroup solution with the image describing each parameter using *normalized* means where 1 = the maximum value and 0 = 0. For ROM, higher values indicate larger ROM, for lumbo-pelvic rhythm (lumbo-pelvic coordination) higher scores indicate a larger percentage of lumbar contribution, for ‘time to max flexion’ larger scores indicate slower movement, for ‘difference at 0^o^ and 20^o^’ lesser scores indicate a lag of pelvic (versus lumbar) movement with the greatest score indicating a lag of lumbar movement

The odds of correct classification for subgroups 1 to 4 were 19.2, 10.4, 29.4 and 28.2 respectively, well above the minimum value of 5 that is suggested to represent high assignment accuracy [27]. Figure 2 uses lumbo-pelvic kinematic parameters, normalised to a 0 to 1 scale, to illustrate differences between subgroups. Figure 3 provides a clinical interpretation of the four subgroups.

### Movement characteristics of the subgroups

**Subgroup 1** was the largest group with 50% of the total cohort (133/266 people) and represented 78% (98/126) of the NoLBP and 25% (35/140) of the LBP groups. This cluster was characterized by the largest trunk ROM with lumbar and pelvic ROM contributing in almost equal parts to trunk flexion, complete relaxation of extensor muscles in full flexion, quicker movement speed and with relatively synchronous movement of pelvic and lumbar spine at the start and also at 20° of movement.

**Subgroup 2** represented 17% and 37% of the NoLBP and LBP groups respectively. Compared to subgroup 1, subgroup 2 had less trunk ROM, higher lumbar and lower pelvic angular inclination with greater activation of lumbar extensor muscles, slower movement and a greater delay of pelvic motion at the start and at 20° of movement, i.e. angular inclination occurred through the lumbar spine first, followed by pelvic movement.

**Subgroup 3** represented 6% and 24% of the NoLBP and LBP groups respectively. Compared to Subgroup 1, Subgroup 3 had markedly less lumbar movement but similar pelvic angular inclination and was different from Subgroup 2 with a reversed pattern of less lumbar and greater pelvic ROM and with greater lumbar extensor activity at the end of flexion than Subgroups 1 or 2. Subgroup 3 was the only group to have delayed lumbar rather than pelvic motion, i.e. angular inclination occurred at the pelvis first, followed then by movement of the lumbar spine.

**Subgroup 4** contained only people with LBP (14% of the total LBP group) and also displayed the smallest trunk and pelvic angular inclination of all subgroups, but with comparable lumbar flexion ROM. Subgroup 4 had the poorest flexion relaxation response (highest amount of lumbar extensor activity in the fully flexed position), slowest movement speed and greatest pelvic delay at 20° of movement (see Fig. 3).

### Between-subgroup differences

Table 2 displays post hoc analysis of between-subgroup differences. Significant differences were seen for age (Subgroup 1 versus Subgroup 3 only, *p* = 0.0049), direction-specific (flexion aggravates, extension eases) pain intensity, activity limitation, percentage of people with leg pain, and for all kinematic parameters, with most *p* values < 0.001.

**Table 2:** Subgroup descriptions and post hoc analysis

	SubGroup 1	SubGroup 2	SubGroup 3	SubGroup 4	Difference between subgroups
Percentage of total cohort (*n* = 266)	50% (*n* = 133)	27.4% (*n* = 73)	15.4% (*n* = 41)	7.1% (*n* = 19)	
Percentage (and number) of people with LBP in each sub group cluster	26.3% (35)	71.2% (52)	82.9% (34)	100.0% (19)	
Posterior probability of belonging to each cluster	0.95 ± 0.10	0.91 ± 0.13	0.97 ± 0.08	0.97 ± 0.11	
Post hoc analysis – demographics					
Age	36.5 *±* 13.6 ^3^	37.5 *±* 13.7	42.1 *±* 14.8	38.1 *±* 13.5	Yes
Sex (female)	60.9%	57.5%	56.1%	47.3%	No
Pain behaviour (for LBP people only)					
Pain intensity using numerical rating scale (0–10 scale)	5.2 *±* 1.4	5.1 *±* 1.3	5.6 *±* 1.8	5.3 *±* 1.5	No
‘Flexion aggravates, Extension eases’ pain score (0–48 scale) ^a^	12.8 *±* 7.3 ^4^	14.5 *±* 8.0 ^4^	16.4 *±* 8.8 ^4^	22.7 *±* 8.4 ^1,2,3^	Yes
Activity limitation (0–100 scale)	31 *±* 17 ^4^	38 *±* 20	42 *±* 22	48 *±* 26 ^1^	Yes
Percentage of LBP people with leg pain ^b^	36.3% ^2,4^	52.0% ^1,4^	21.8% ^4^	76.5% ^1,2,3^	Yes
Lumbo-pelvic flexion kinematic parameters					
Trunk Peak ROM (^o^)	111 *±* 12 ^2,3,4^	97 *±* 17 ^1,3,4^	89 *±* 16 ^1,2,4^	77 *±* 20 ^1,2,3^	Yes
Lumbar Peak ROM (^o^)	51 *±* 9 ^3^	54 *±* 10 ^3,4^	30 *±* 8.5 ^1,2,4^	47 *±* 14 ^2,3^	Yes
Pelvic ROM (^o^)	60 *±* 11 ^2,4^	44 *±* 5 ^1,4^	59 *±* 15 ^2,4^	31 *±* 11 ^1,2,3^	Yes
Percentage of lumbar contribution to trunk flexion (%)	47 *± 7* ^2,3,4^	57 *±* 10 ^1,3^	35 *±* 9 ^1,2,4^	60 *±* 9 ^1,3^	Yes
Flexion relaxation response	0.00 *±* 0.00 ^2,3,4^	0.04 *±* 0.05 ^3,4^	0.48 *±* 0.50 ^1,2^	0.72 *±* 0.55 ^1,2^	Yes
Duration of trunk flexion (sec)	2.31 *±* 0.63 ^2,3,4^	3.11 *±* 1.11 ^1^	2.87 *±* 0.70 ^1^	4.10 *±* 1.83 ^1^	Yes
Pelvic time-lag at start of movement (sec)^c^	+ 0.17 *±* 0.14 ^2,4^	+ 0.42 *±* 0.31 ^1,3^	+ 0.13 *±* 0.24 ^2,4^	+ 1.10 *±* 1.34 ^1,3^	Yes
Pelvic time-lag at 20^o^ of movement (sec)^c^	+ 0.22 *±* 0.30 ^2,4^	+ 0.86 ± 0.53 ^1,4^	**-** 0.55 *±* 0.8 ^4^	+ 2.04 *±* 1.74 ^1,2,3^	Yes

Superscript numbers represent subgroups i.e. ^3^ = Subgroup3 and indicate a significant difference between the column named subgroup and the superscripted subgroup

^a^A study-specific, non-validated questionnaire based on directional pain responses where flexion aggravates and extension eases (see Appendix)

^b^Percentage calculated by number of people with leg pain in each subgroup over number of people with LBP in each subgroup

^c^positive numbers indicate a time-lag (delay) of pelvic movement, i.e. the lumbar spine moves first then the pelvis begins to move, lagging behind lumbar movement (at start and at 20^o^ of lumbar and pelvic flexion). Negative numbers indicate a time-lag for the lumbar spine, i.e. the pelvis moves or achieves 20^o^ of flexion earlier than the lumbar spine achieving 20^o^

## Discussion

This study used data from a previous observational cohort study to examine whether patterns of movement could be seen in multivariable flexion-related lumbo-pelvic kinematics (eight standing flexion parameters and three sitting parameters) and if these patterns occurred equally in people with and without persistent LBP. Latent Class Analysis identified four relatively well-defined subgroups with three of the subgroups containing both NoLBP and LBP participants, and one subgroup consisting of LBP participants only. These results support the concept that people demonstrate heterogenous movement characteristics, and some of those patterns are associated with persistent LBP. These findings align with the heterogeneity reported in and across other health data such as cognitions, pain behaviour, and improvement trajectories.

The concept of movement-related subgroups is not new. Two of the movement patterns identified in this sample are similar to patterns described in other classification systems such as the flexion and ‘active-extension’ motor control impairment described by O’Sullivan [14, 21] with Subgroup 2 and Subgroup 3 respectively matching these descriptive groups. Several studies using pre-classified groups have identified kinematic differences between flexion and ‘active extension’ subgroups, and between people with LBP and healthy controls [21, 28–30]. However, in all of these studies, subgroups were pre-defined based on observation and history, without objective measurement of lumbo-pelvic kinematics, and analysed smaller samples. Where studies subsequently contrasted those subgroups using laboratory-based measurement tools, these contrasts were usually only univariate comparisons. This study differs by using multivariable clusters of lumbo-pelvic kinematic parameters to describe patterns that are seen in both NoLBP and LBP populations, in a large sample using wireless motion and surface EMG sensors that are readily available for clinical settings.

### The relationship between movement and pain

Subgroups 1, 2, and 3 all included people who reported never having had LBP that warranted seeing a clinician or taking time off work or sport. The presence of people with no history of LBP in these subgroups, particularly Subgroups 2 and 3, suggest that these movement patterns can pre-exist injury or a chronic pain experience. The decreasing percentage of people with no LBP history within Subgroups 2–4 suggests that pain and movement are associated, and that identifying cause and/or consequence relationships between pain and movement is likely to be important. Subgroup 4 included only people from the LBP group. The observed reduced movement range and increased muscle activation may be protective of, or a reactive response to, pain. However, we do not know if pre-existing movement patterns, such as those seen in Subgroups 2 and 3, increase the risk of developing LBP. Further research is required to see if the presence of a particular movement pattern or specific lumbo-pelvic kinematic parameter increases the risk of LBP occurrence, delays recovery or is associated with differing trajectories of recovery.

The mean pain score did not differentiate between subgroups, a finding previously seen in other subgrouping studies [29]. However, direction-specific pain questions (does flexion aggravate and extension ease pain?) showed increasing pain scores with correspondingly reduced ROM from Subgroups 1 to 4 and increasingly reduced flexion relaxation. Clinicians often observe a pain response matched to directionally specific movement ([13, 31, 32], so this relationship between flexion aggravation pain scores and flexion kinematics is not surprising. A similar pattern of progressively increased activity limitation from Subgroups 1 to 4 was seen and is consistent with the direction-specific pain score that quantified flexion-related pain activities. Leg pain and pelvic ROM also showed the interesting and clinical plausible finding where the two subgroups that had the lowest pelvic ROM also had the largest percentage of people with a leg pain component associated with their LBP (52% and 76% for Subgroups 2 and 4 compared to 36% and 22% for Subgroups 1 and 3).

### Implications for research and clinical management

The presence of relatively distinct and different patterns lends support to the concept that treatments are likely to be more effective if the treatment matches the identified deficit. For example, improving the flexion relaxation response is recommended for people with persistent LBP and may be helpful for people in Subgroups 3 and 4 but is unlikely to assist when people with persistent LBP have the flexion movement pattern seen in Subgroups 1 and 2. Similarly, improving lumbar ROM may be helpful for people in Subgroup 3, where lumbar flexion has the greatest reduction, but is less likely to be useful for people in Subgroup 4 where lumbar flexion is only slightly less than almost 80% of the NoLBP group. While there is limited evidence that individualized treatment approaches have favourable outcomes [31, 33–35], it is unknown if treatments aimed at specific kinematic subgroups have better outcomes. If these subgroups continue to be seen in other samples, matching specific treatments to subgroups based on lumbo-pelvic kinematics could be a focus for further research.

While pain and activity limitation are seen to some extent in all people with persistent LBP, this is not necessarily true for the presence of some lumbo-pelvic kinematic features. In this sample, 25% of people with persistent LBP had a ‘standard’ pattern of movement that was found in almost 80% of the NoLBP group, suggesting that people in this subgroup have flexion kinematics that are not obviously affected by pain and are the same as people without LBP. It is possible that other unmeasured parameters (e.g. ROM in other directions, different muscle activation patterns or strength factors) might have been problematic or it may be that movement factors are not relevant for some people with persistent LBP. This has implications for research and measuring change in movement as an outcome measure. Measuring changes to pain and activity limitation are relevant to most LBP patients but measuring change to movement may be less relevant for some people.

### Strengths

Classification accuracy was high which provides greater confidence in observing subgroup patterns. The sample size was sufficiently large to observe non-predetermined patterns. An additional benefit was the inclusion of 126 people with no history of significant back pain which allowed insight into whether movement patterns could pre-exist the onset of pain.

There are clinically relevant strengths of this study. The use of single, univariable comparisons has frequently been used to contrast NoLBP and LBP groups, with varying results [18]. A strength of using multivariable lumbo-kinematic parameter analysis that uses clusters of parameters to define patterns (subgroups) of patients is that it reflects real-world clinical practice which incorporates many sources of information in decision-making. For example, including pelvic ROM as one of the flexion-related lumbo-pelvic parameters combined with the flexion relaxation response helped differentiate between Subgroups 2 and 4. Conversely, if lumbar ROM were the main measure of physical assessment without reference to other measures, the distinction between those subgroups would not be possible. Another clinically relevant strength is that the lumbo-pelvic kinematic parameters used in this study can all be measured in a typical clinical setting.

### Limitations

Flexion was chosen as the focus of kinematic assessment because flexion-related activities have been previously identified as the most common pain-related activities in people with LBP [17]. Additionally, previous work has shown that flexion has greater measurement reliability and consistency compared to other directions, most likely due to the larger relative ROM, limited effect of attenuation of range on correlational indices, and lower susceptibility to skin movement artefacts [23]. However, other movement directions and parameters (i.e strength, proprioception) may also inform clinical decision-making. The inclusion of other movement-related parameters are likely to add to, and change, overall subgroup profiles. It is also possible that while flexion was not problematic for some of the people with persistent LBP in this sample, other movement directions, e.g. extension, could have been painful for them. Also, functional tasks are often three dimensional, whereas this sample of people were tested using sagittal plane motion only. However, Marras et al. [36] and Gombatto [28] both assessed para-sagittal and three-dimensional movement, with both studies demonstrating that the sagittal plane was the movement plane where movement effects were most visible. It would both be very difficult to assemble a sample of people who had never experienced any LBP at any time point, and the results from such a group would not be broadly applicable to the general population. In addition, age can affect ROM and there was a significant difference in age only between Subgroups 1 and 3 of approximately 6 years. In our view, that difference is unlikely to account for the 21^o^ difference of lumbar ROM seen between those subgroups. Another limitation of the study was that other pain-related parameters such as duration of pain and frequency of recurrence may have provided additional information about subgroup characteristics. Lastly, these results have not been verified in an independent sample and, until such time, the possibility that observed clusters are sample specific, must be considered.

## Conclusion

Movement was studied in 140 people with and 126 people without persistent LBP, with four movement-pattern subgroups seen in flexion related lumbo-pelvic kinematics. Subgroup 1, the ‘standard’ group was the largest, accounting for almost 80% of NoLBP and 25% of people with LBP and 50% of the total group. Subgroup 1 (‘standard’ subgroup) had the greatest trunk ROM, full flexion relaxation at end range flexion, and relatively synchronous pelvic and lumbar movement. Subgroups 2 (‘lumbar-dominant’) and 3 (‘pelvic-dominant’) showed progressive loss of flexion relaxation and opposite lumbo-pelvic rhythm patterns. Subgroup 4 (‘guarded’ movement) had the lowest trunk and pelvic ROM, but similar lumbar ROM to the standard subgroup, had the highest extensor muscle activation in full flexion, the slowest movement, and the greatest pelvic delay. In addition, leg pain occurred more frequently in the two subgroups that had the lowest range of pelvic movement. Although mean pain intensity scores were similar across subgroups, activity limitation and the ‘flexion aggravates/extension eases’ pain scores progressively increased, reaching significance for the comparison between Subgroup 1 (standard) and Subgroup 4 (guarded). These results indicate that different patterns of flexion are present in people with and without persistent LBP and this has implications for both further research and treatment.

### Additional file

Additional file 1: Definition details for lumbo-pelvic kinematic parameters. (DOCX 22 kb)

## Figures and Tables

**Fig. 4:**
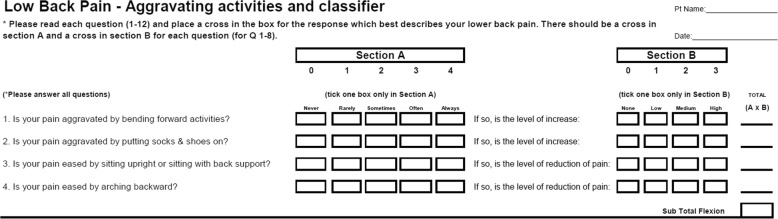
The study-specific, non-validated ‘does flexion aggravate and extension ease’ (FLAG) pain questionnaire

**Fig. 5:**
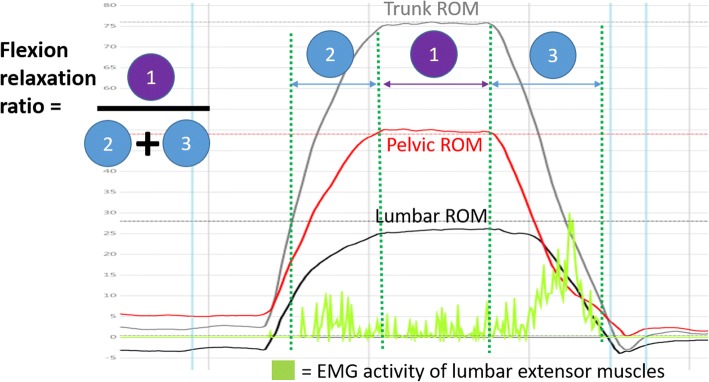
Calculation of the flexion relaxation response. Displays a person moving into flexion with the X axis representing time and the Y axis representing ROM. The green line indicates EMG activity of lumbar extensors muscles. The calculation for determinig the flexion relaxation ratio is displayed

**Fig. 6:**
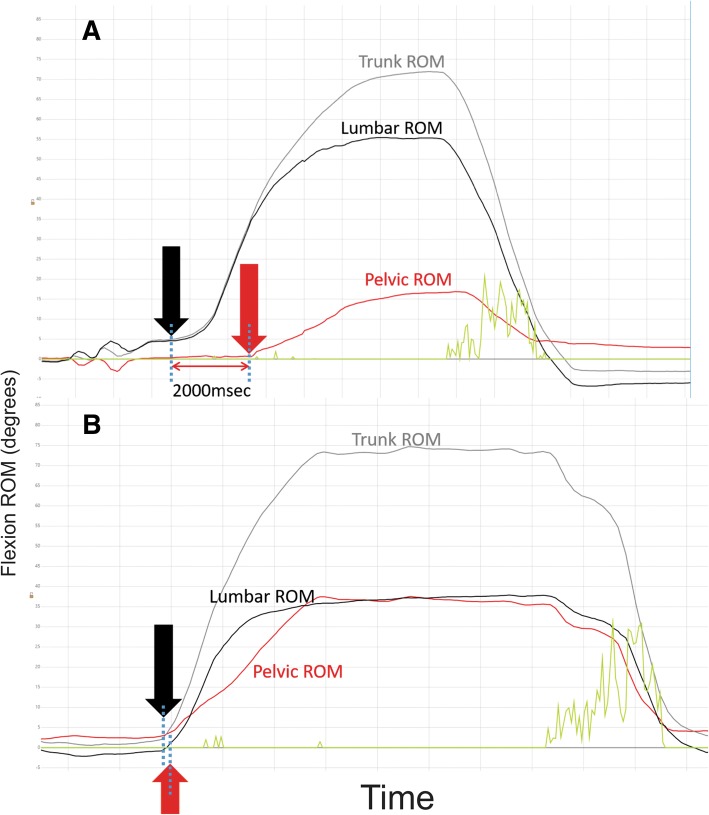
Onset delay of lumbar or pelvic movement. These graphs show ROM (Y axis) changes over time (X axis). Graph A was from a person who moved their lumbar spine into flexion with a two second delay before the pelvis started moving. Graph B shows a more typical pattern with a synchronous start of movement of the lumbar spine and pelvis. The green line indicates EMG activity of lumbar extensor muscles

## Abbreviations

FRR: Flexion relaxation response
LBP: Low back pain
NoLBP: Participants without low back pain
RMDQ: Roland Morris disability questionnaire
ROM: Range of motion
