# Supplementary material for: Subgroups of lumbo-pelvic flexion kinematics are present in people with and without persistent low back pain
Source: BMC Musculoskelet Disord. 2018 Aug 28;19:309. doi: 10.1186/s12891-018-2233-1 (PMC6114878; doi:10.1186/s12891-018-2233-1)
Supplement: Supplementary file 1 — Definition details for lumbo-pelvic kinematic parameters. (DOCX 22 kb) [file 12891_2018_2233_MOESM1_ESM.docx]

# Additional file 1

## Definition of kinematic characteristics

### Range of motion (ROM)

Trunk ROM was measured as angular inclination of the trunk at T12, pelvic ROM was measured as angular inclination of the pelvis at S2 and lumbar ROM was calculated using the difference between the angular inclinations at T12 and S2.

### Lumbo-pelvic Coordination (rhythm)

Lumbo-pelvic coordination, sometimes described as lumbo-pelvic rhythm, is a method of describing lumbar versus pelvic contributions to movement. We calculated the relative contribution of lumbar movement at the end range of trunk flexion by using lumbar peak angle divided by trunk peak angle and expressed as a percentage.

### Flexion relaxation response

A common pattern of thoraco-lumbar extensor muscle activity measured by surface electromyography (sEMG) is seen in people without back pain with electrical activity occurring at the start of trunk flexion (eccentric activation) and again on return from the fully flexed position (concentric activity), with minimal or no activity in the fully flexed position. This has been described as the flexion relaxation response [1]. Flexion relaxation is often absent in people with LBP when compared to people without LBP, and when restored, is associated with improvements in pain and activity limitation [2, 3]. It is possible that higher extensor muscle activity in the fully flexed position, a position that is recognized as a biomechanically vulnerable position for the intervertebral disc [4], increases compressive loading. The flexion relaxation response was calculated as the sum of sEMG activity (millivolts) during 3 seconds in the fully flexed position (numerator) divided by the summed sEMG activity during both the eccentric (forward bending) and concentric (returning to upright stance) phases of flexion (denominator). The ‘normal’ complete muscle relaxation in full flexion would result in this flexion relaxation response being reported as close to or equal to zero. As muscle activity in end of range flexion increases, this ratio increases, with a larger number indicating greater muscle activation in the fully flexed position. Figure 5 provides a visual explanation of the FRR calculation.

### Figure 5 placed HERE

### ‘Onset delay’ and ‘Delay-at-20^o^’ of trunk flexion

Because motion sensors measure movement over time, it is possible to assess time-related synchronicity of lumbar versus pelvic contributions to flexion movement. There is evidence of time-related differences in lumbar versus pelvic movement during flexion [5]. An ‘onset-delay’ parameter measures which region, lumbar or pelvis, moves first and the time ‘gap’ between regions. Negative numbers indicate a delay in pelvic motion, with movement initiated first in the lumbar spine, while positive numbers indicate a delay in lumbar motion, with movement initiated at the pelvis. Larger numbers indicate a longer delay. The start of flexion was defined as the point at which velocity was > 7^o^/sec (the velocity required before movement was visible graphically). Figure 6 demonstrates an example of an onset-delay in pelvic movement. The ‘delay-at 20^o^’ parameter provides a similar view of movement discrepancy and is a calculation of the time needed to achieve 20^o^ of angular inclination from the start of movement, for each region. Both parameters provide a measure of time-related synchronicity (or lack thereof) of lumbar versus pelvic contribution to flexion. The time difference in lumbar versus pelvic movement achieving 20^o^ of angular inclination was chosen as almost all participants produced a reading of 20^o^ for both lumbar and pelvic movement, whereas at 30^o^ and 40^o^, 13% and 33% of participants respectively did not achieve these angles for either lumbar or pelvic motion.

###

**Figure 6 placed HERE**

### Flexion movement duration

Flexion movement duration was defined as the time taken from start of trunk flexion (when velocity of movement was >7°/sec) to the fully flexed position (when velocity was <7°/sec velocity). We defined end of trunk flexion in this way because movement with a velocity less than 7^o^/sec is very close to end-range and this threshold minimizes error that can result from the peak angle slowly increasing due to creep when the fully flexed position is sustained for the three second period during which we assessed the flexion relaxation response.

### Sitting: Pelvic tilt range and pelvic tilt ratio

Pelvic tilt ROM (from full posterior to anterior tilt angular inclination) may be of clinical interest when sitting is associated with pain. Reduced pelvic repositioning accuracy (proprioception) and reduced movement variability have been identified in people with chronic LBP [6-10]. The pelvic tilt range was measured by calculating the angular inclination of the pelvis between full anterior and full posterior tilt, which provided estimates of lower lumbar movement. The pelvic tilt ratio is a measure of the independence of pelvic tilt relative to trunk movement and is calculated by dividing the angular inclination of the pelvic sensor by the angular inclination of the trunk sensor. This parameter was used to test how pelvic tilting was performed i.e. whether movement was independently performed only in the lower lumbar motion or combined with upper lumbar motion, as might occur if a person simultaneously moved the trunk into flexion while performing posterior pelvic tilt). A number > 1 indicates larger pelvic than trunk ROM; a number <1 indicates larger trunk than pelvic ROM during the pelvic tilt manoeuvre.

### Sitting: relative position

Measurements were made of usual, full slumped (kyphotic) and full upright (lordotic) sitting lumbar positions. The relative sitting position was calculated for usual sitting by deeming the fully slumped sitting position to be 100% and the fully upright sitting to be 0%. For example, if full slump was at 50^o^ of lumbar flexion and full upright sitting was at 0^o^ lumbar flexion, then the difference (50^o^-0^o^=50^o^) between maximum slump and upright sitting would represent 100% of the available ROM. If usual sitting was 25^o^, the relative sitting position would have been coded as 50%. This index enabled comparisons between individuals for defining usual sitting position relative to the available range of pelvic movement.

Bibliography

1. Floyd W, Silver P: **The function of the erectores spinae muscles in flexion of the trunk**. *Lancet* 1951, **6647**:123-133.

2. Geisser ME, Ranavaya M, Haig AJ, Roth RS, Zucker R, Ambroz C, Caruso M: **A meta-analytic review of surface electromyography among persons with low back pain and normal, healthy controls**. *J Pain* 2005, **6**(11):711-726.

3. Neblett R, Mayer TG, Brede E, Gatchel RJ: **The effect of prior lumbar surgeries on the flexion relaxation phenomenon and its responsiveness to rehabilitative treatment**. *The Spine Journal* 2014, **14**:892-902.

4. O'Connell GD, Vresilovic EJ, Elliott DM: **Human intervertebral disc internal strain in compression: The effect of disc region, loading position, and degeneration**. *Journal of Orthopaedic Research* 2011, **29**(4):547-555.

5. Wong TK, Lee RY, Wong TKT, Lee RYW: **Effects of low back pain on the relationship between the movements of the lumbar spine and hip**. *Human Movement Science* 2004, **23**(1):21-34.

6. Laird R, Gilbert J, Kent P, Keating J: **Comparing lumbo-pelvic kinematics in people with and without back pain: a systematic review and meta-analysis**. *BMC Musculoskelet Disord* 2014, **15**(1):229.

7. Hodges PW, Coppieters MW, MacDonald D, Cholewicki J: **New insight into motor adaptation to pain revealed by a combination of modelling and empirical approaches**. *European Journal of Pain* 2013, **17**(8):1138-1146.

8. Seay JF, E.A, Hamill J: **Low back pain status affects pelvis-trunk coordination and variability during walking and running**. *Clin Biomech* 2011, **26**(6):572-578.

9. Abboud J, Nougarou F, Page I, Cantin V, Massicote D, Descarreaux M: **Trunk motor variability in patients with non‑specific chronic low back pain**. *European Journal of Applied Physiology* 2014.

10. Villumsen M, Madeleine P, Jorgensen M, Holtermann A, Samani A: **The variability of the trunk forward bending in standing activities during work vs. leisure time**. *Applied Ergonomics* 2016, **58**:273-280.
